# Photocatalytic Water Splitting—The Untamed Dream: A Review of Recent Advances

**DOI:** 10.3390/molecules21070900

**Published:** 2016-07-09

**Authors:** Tahereh Jafari, Ehsan Moharreri, Alireza Shirazi Amin, Ran Miao, Wenqiao Song, Steven L. Suib

**Affiliations:** 1 Institute of Materials Science, University of Connecticut, 91 North Eagleville Road, Storrs, CT 06269-3222, USA; tahereh.jafari@uconn.edu (T.J.); ehsan.moharreri@uconn.edu (E.M.); 2 Department of Chemistry, University of Connecticut, 55 North Eagleville Road, Storrs, CT 06269-3060, USA; alireza.shirazi_amin@uconn.edu (A.S.A.); ran.miao@uconn.edu (R.M.); wenqiao.song@uconn.edu (W.S.)

**Keywords:** water splitting, solar fuels, hydrogen, photocatalysis, photocatalysts, semiconductors, nanomaterials, metal oxides, nanotechnology

## Abstract

Photocatalytic water splitting using sunlight is a promising technology capable of providing high energy yield without pollutant byproducts. Herein, we review various aspects of this technology including chemical reactions, physiochemical conditions and photocatalyst types such as metal oxides, sulfides, nitrides, nanocomposites, and doped materials followed by recent advances in computational modeling of photoactive materials. As the best-known catalyst for photocatalytic hydrogen and oxygen evolution, TiO_2_ is discussed in a separate section, along with its challenges such as the wide band gap, large overpotential for hydrogen evolution, and rapid recombination of produced electron-hole pairs. Various approaches are addressed to overcome these shortcomings, such as doping with different elements, heterojunction catalysts, noble metal deposition, and surface modification. Development of a photocatalytic corrosion resistant, visible light absorbing, defect-tuned material with small particle size is the key to complete the sunlight to hydrogen cycle efficiently. Computational studies have opened new avenues to understand and predict the electronic density of states and band structure of advanced materials and could pave the way for the rational design of efficient photocatalysts for water splitting. Future directions are focused on developing innovative junction architectures, novel synthesis methods and optimizing the existing active materials to enhance charge transfer, visible light absorption, reducing the gas evolution overpotential and maintaining chemical and physical stability.

## 1. Introduction

The continual increase in world population and lifestyle standards has led to a seminal growth in global energy consumption [1]. Amounting to about 90% of global energy, fossil fuels supply the transportation and industrial sectors, leading to high emission of greenhouse gases including carbon dioxide [2,3] and resulting in a substantial depletion of carbon-based resources that could be otherwise used for the production of valuable chemicals. In 2013, worldwide energy consumption was 17 TW and is expected to at least double by 2050 [4]. Development of a clean and renewable source of energy is crucial to mitigate consequences of fossil fuel consumption including climate change, eventual depletion of energy supplies, market uncertainty, and foreign oil dependency [5,6,7].

There are several alternative energy sources including wind, geothermal, hydropower, and solar which are relatively clean and sustainable in comparison with fossil fuels, however, each of them has some limitations which make this substitution challenging. Electricity generated by wind turbines is not storable. Hydropower suffers from dam construction limitations due to high cost and possible adverse environmental effects. Geothermal energy is a continuous source which is limited in lifetime and is subsequently costly in operation [8].

Being unlimited, renewable and free, solar energy is capable of producing electricity or heat without the requirements of having turbines and maintenance. The energy usage of one year could be provided by half an hour of solar irradiation on the Earth surface [9]. However, sunlight is an intermittent source of energy which limits the amount of solar radiation due to its dependence on geographical position, day, time, and season [10,11]. Another disadvantage of solar energy is its low density per unit of Earth surface [12]. Therefore, developing a source of energy which is storable, clean, continuous and renewable, is required to meet global energy demand. Hydrogen is an advantageous fuel for being: (1) abundant from various sustainable sources (biomass or water); (2) having high energy yield; (3) environmentally friendly and (4) high storage capability, thus it is considered as an ideal alternative source of energy for fossil fuels [13,14,15].

### 1.1. Hydrogen and Related Concerns

Although hydrogen with its unique properties of high-energy efficiency, easy storage, and freedom from pollution has been considered as a promising alternative to the conventional sources of energy, H_2_ has some drawbacks, which need to be addressed for it to be practically used as fuel. Storing hydrogen as a compressed gas or liquid requires energy and additional costs [16]. The limited infrastructure for hydrogen fueling is another factor limiting its practical use. The most important hitch of current hydrogen production methods is the reliance on fossil fuels (natural gas reforming) for its production. Extensive research has therefore been conducted to explore techniques for producing hydrogen from renewable sources.

### 1.2. Hydrogen Evolution by Solar Energy

Steam methane reforming is a widely used technique to produce hydrogen from natural gas at high temperatures (up to 900 °C) and pressures (1.5–3 MPa) [17,18]. Coal gasification is also employed to generate hydrogen through partial oxidation at high temperatures and pressures (up to 5 MPa) [17]. Biomass materials such as crops, animal wastes, and plants under thermochemical routes generate hydrogen through pyrolysis and gasification which produce byproducts of CO, CO_2_, and methane [19]. Biological processes for hydrogen production from biomass materials are other promising techniques [20] but they are not economically feasible yet. Consequently, current hydrogen generation techniques suffer from a reliance on fossil fuel sources, harsh process conditions, and significant costs. Alternative methods that utilize renewable sources of energy for hydrogen production such as hydropower, wind power, and sunlight must be explored. Among these sustainable energies, solar energy has been considered a more promising source due to its lesser location dependence in comparison to wind and hydropower energy.

The combination of solar energy with plentiful water resources provides a reasonable platform for hydrogen generation which is called solar water splitting [21,22,23]. There are three approaches to split water using solar energy [24]: (1) thermochemical water splitting; (2) photobiological water splitting; and (3) photocatalytic water splitting. Although the thermochemical approach is the simplest, the requirement for large solar concentrators makes this method highly expensive and less favorable [25]. Biophotolysis can be divided into water biophotolysis and organic biophotolysis depending on the microorganism type, product, and mechanisms of the reaction [26]. Although water biophotolysis is cleaner than organic biophotolysis (regarding CO_2_ emissions) [27], low yields of hydrogen production, toxic effects of enzymes, and limitations on scaling up the process exist [28]. Photocatalytic water splitting possesses several advantages over thermochemical and photobiological water splitting including: (1) low cost (capable of reducing the photovoltaic arrays) [29]; (2) relatively high solar-to-H_2_ efficiency; (3) capability of separating H_2_ and O_2_ streams; and (4) flexible reactor size which is appropriate for small scale usage [30]. The US Department of Energy (DOE) has established the ultimate target of 26% for the solar to hydrogen energy conversion ratio which requires aggressive research to improve the current status [31].

### 1.3. Photocatalytic Water Splitting

To achieve overall water splitting and investigate structure-property relationships of photocatalysts the two half reactions of water splitting have been studied extensively. These reactions being hydrogen and oxygen evolution reactions usually involve the use of sacrificial reagents to improve the hydrogen and oxygen yield. Even though a catalyst can catalyze both reactions with the aid of sacrificial electron donors and acceptors, this may not work for overall water splitting. To clarify, water splitting discussed in this review refers to directly splitting of water into hydrogen and oxygen in a 2:1 ratio by the use of a proper photocatalyst. Several research and review articles have proposed the mechanisms of photocatalytic water splitting [32,33,34]. The reaction is first initiated by photon absorption, which generates numerous electron-hole pairs with sufficient potentials. Those charge carriers then migrate to the surface of the catalysts and react with surface active sites. Finally, the photo-generated electrons reduce water to form hydrogen, and the holes oxidize water molecules to give oxygen.

Fujishima and Honda first reported the overall photocatalytic water splitting by a titanium dioxide (TiO_2_) electrode [35]. Since this pioneering work, numerous research studies of water splitting have been conducted on semiconductor materials, especially via heterogeneous catalysis. Semiconductors have non-overlapping valence bands and conduction bands with a band gap in between that of insulators and conductors. When sufficient photochemical energy is applied, electrons will be excited into the conduction band, leaving electron holes in the valence band and excess electrons in the conduction band. These electron-hole pairs play key roles in the redox reactions of water splitting. Electrons are responsible for reducing protons to hydrogen molecules, and oxygen anions will be oxidized by the holes. In order to initiate the redox reaction, the highest level of the valence band should be more positive than water oxidation level ($E_{O_{2}/H_{2}O}$, 1.23 V vs. Normal hydrogen electrode; NHE), while the lowest level of the conduction band should be more negative than the hydrogen evolution potential ($E_{H_{2}/H_{2}O}$, 0 V vs. NHE).

(1) H2O+2h+→2H++12O2                        Eoxidationo=−1.23 V

(2) $$2H^{+}+2e^{-}\to H_{2}\;E_{reduction}^{o}=0.00\;V$$

(3) H2O→H2+12O2

Therefore, the minimum band gap for a suitable water splitting photocatalyst should be 1.23 eV. Accordingly, TiO_2_, ZrO_2_, KTaO_3_, SrTiO_3_, and BiVO_4_ are good candidates for photocatalytic water splitting [36,37,38] However, some typical semiconductors such as SiC, ZnO, and CdS, even though their band gap fits well into the water splitting redox potential, are not active for water splitting due to photocorrosion. Photo-corrosion happens when the anion from the catalyst itself is oxidized by photogenerated holes instead of water. Another challenge is that most semiconductor catalysts only operate under ultraviolet (UV) light which accounts for only ca. 4% of the total solar energy [32,33,34]. To improve the solar energy efficiency, photocatalysts with the ability to work under visible light are highly desirable, since visible light contributes to almost half of the incoming solar energy. The band gap of semiconductor materials should be less than 3 eV to have a visible light response. Recently, semiconductor catalysts coupled with carbon materials or precious metal particles have been shown to have better visible light response [35,36]. In addition, metal sulfides, metal nitrides, and metal-free catalysts are also promising catalytic systems for photocatalytic water splitting by visible light [37,38,39,40].

Traditional water-splitting photocatalysts are based on transition metal oxides which form stable compounds due to the high electronegativity of oxygen atoms [41]. Transition metal oxides can be classified into two groups according to their d orbital structure. Early transition metals like Ti, V, Nb, and W have empty d orbitals, thus having a low valence band energy. Also, their valence bands are strongly influenced by the oxygen 2p orbitals. As a result, these materials have large band gaps which make them less efficient for photocatalytic reactions. Several strategies including doping and creating defects have been engaged to increase their light absorption efficiency. For example, Zhao et al. successfully designed defect-enriched black TiO_2_ through high-temperature hydrogenation and the synthesized material exhibited excellent photocatalytic hydrogen evolution reactivity [42]. On the other hand, late transition metals such as Mn, Fe, Co, and Ni have occupied d orbitals. Their oxides usually have small band gaps and the strong d-d transitions play significant roles [41]. Fe_2_O_3_ is a typical example in this group due to its abundant and inexpensive nature, and its attractive photocatalytic activities have been reported [43,44]. Having little polaron conductivity is the disadvantage of late transition metal oxides [45]. To overcome those limitations, multicomponent metal oxides have been developed. Moreover, metal nitrides and metal sulfides were synthesized and shown better photocatalytic activities. Wei et al have reported that combining cations with s^2^ and d^0^ configurations can lower the band gap. The coupling between s band from s^2^ cation and p band from oxygen can increase the valence band level while the coupling between d band from d^0^ cation and p band can lower the conduction band level [46]. A typical example of this type of ternary oxides is BiVO_4_. Its photocatalytic properties have been intensively studied over the years [47,48,49,50]. Further doping BiVO_4_ with other cations such as Ag^+^, V^5+^, and W^6+^ can increase its electronic conductivity and resulting in better catalytic performance [51,52,53]. Other examples of band gap tuning by ternary oxides include CuWO_4_, ZnFe_2_O_4_, CaFe_2_O_4_, CuBi_2_O_4_, and CuNb_3_O_8_, etc. [54,55,56,57,58]. Besides metal doping techniques; nitrogen substituting can also decrease the band gap due to its higher-lying 2p orbital levels [59,60]. Like nitrogen, sulfur and selenium also possess higher-lying p bands than those of oxygen; they can also be used to create smaller band gap materials than their oxides counterparts [61,62,63]. Moreover, modification of the catalysts with silicon, group III-V semiconductors, and carbon-based materials have been reported and proved to be efficient methods for developing photoactive materials [64,65,66]. The summary of very recent photocatalysts is presented in Table 1. It is crucial to note that the amount of active photocatalyst material, the light source, turnover frequency and catalytic stability is different in each entry of the table. Therefore, the hydrogen production should not be deemed as the sole measure of performance in every system.

Once the electron-hole pairs are generated, these charge carriers need to move to the surface of the catalysts and catalyze water splitting at the interfaces between the electrode and electrolyte. The major challenge in this step is the recombination of electrons and holes [32,34]. The photogenerated electron-hole pairs can recombine in a short period of time before they catalyze the redox reactions, releasing heat or photon energy. In general, fewer defects and small particle size are believed to be able to inhibit the recombination of electrons and holes. Surface defects usually can serve as adsorption sites for electrons and holes and facilitate their recombination before redox reaction, thus decreasing photocatalytic activity. Highly crystalline and stoichiometric materials have fewer defects on the surface; therefore, they are beneficial for the overall water splitting reaction. On the other hand, nanosized materials can provide short diffusion distances for electrons and holes to get to the surface active sites, thus limiting the recombination probability. Nonetheless, materials with small particle size usually lead to high surface area, which contributes to effective interaction between charge carriers and surface active sites.

Lastly, the migrated electrons and holes will interact with surface active sites and go through a series of redox reactions to produce hydrogen and oxygen. At this point, the intrinsic activity and the number of the surface active sites become crucial. Even if the photogenerated electrons and holes are well separated and reached the material surface, the reaction cannot happen without proper active sites. The bottom level of conduction bands of many transition metal oxides are not negative enough to start the hydrogen evolution reaction, so co-catalysts such as precious metals and NiO are needed to provide assistance for water reduction [32]. However, the top level of valence bands of metal oxides are usually positive enough to oxidize water to oxygen without the aid of co-catalysts. High surface areas can provide more accessible active sites and are reported to be beneficial for the water splitting reaction but, this factor is not as large as other structural parameters such as crystallinity and particle size [67,68,69,70]. This is due to the adsorption of reactant water molecules not being that dominant in water splitting as in other reactions like dye degradation. Moreover, water splitting into hydrogen and oxygen is an energy demanding reaction, which is thermodynamically unfavorable. The backward reaction is more likely to occur. Therefore, the separation and removal of produced oxygen and hydrogen play a major role in this reaction.

## 2. Photocatalytic Reactions

### 2.1. Types of Reaction

#### 2.1.1. Photochemical Reactions

**Figure 1:**
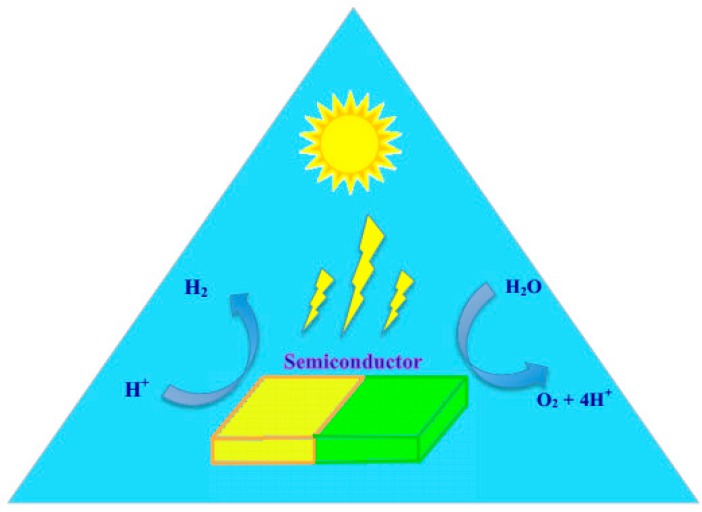
Schematic representation of photochemical water splitting. Figure adapted from reference [90] of Currao work.

Heterogeneous photochemical water splitting consists of three components: a catalyst, visible light absorber, and sacrificial electron donor. Although the basic principles of photochemical and photoelectrochemical systems are identical, they differ in their setup. In photochemical reactions, there is a semiconductor-electrolyte junction at which the water splitting reaction takes place (Figure 1). The required potential for water splitting is generated at the semiconductor-liquid interface. The semiconductor should be stable in the electrolyte to prevent any corrosion. Depending on the band edge position of the semiconductor as discussed previously, they can be active in hydrogen production, oxygen production, or overall water splitting [89].

#### 2.1.2. Photo-Electrochemical Reactions

**Figure 2:**
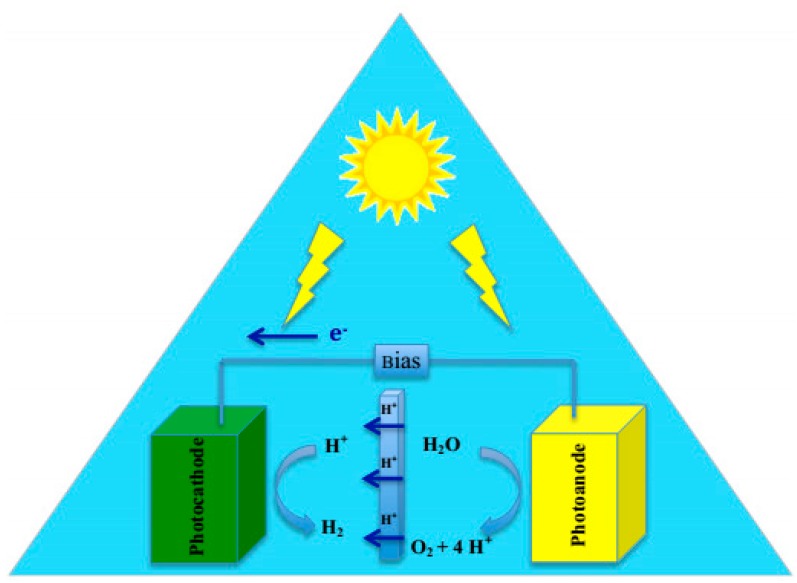
Schematic representation of photoelectrochemical water splitting, Figure adapted from reference [90] of Currao work.

In photoelectrochemical (PEC) water splitting, a photocatalyst, which is a semiconductor, is irradiated by UV-visible light with energy greater or equivalent to the band gap of the semiconductor (Figure 2). The light energy will be absorbed by the photocatalyst and results in charge separation at the valence band and conduction band. The holes are produced at the valence band, and the photo-excited electrons are located in the conduction band. The holes trigger the oxidation of water at the surface of conduction band while the photo-excited electrons at conduction band reduce the absorbed H^+^ to H_2_. Mainly in photoelectrochemical water splitting, semiconductors are applied as a photocathode or photo-anode depending on the reaction, which is favored. In photo-electrochemical water splitting, a semiconductor electrode should be in contact with an electrolyte, which contains a redox couple. In PEC water splitting, the overall reaction takes place at two different electrodes. In this method, the potential which is needed for water splitting is being provided by illuminating the cathode or anode [91].

### 2.2. Reaction Setup

The most common experimental setup used by researchers consists of a reaction cell, a gas circulation pump, vacuum pumps, and a gas chromatograph detector. The oxygen and hydrogen produced can also be detected using oxygen and hydrogen sensors, or by volumetric methods. The reaction solution should be purged with inert gases before testing, and the whole setup should be air-free to measure the amount of evolved oxygen accurately. Several light sources can be used to initiate the reaction. For photocatalysts with a UV light response, high-pressure mercury lamps are employed and the reaction cell should be quartz. For the catalysts with band gaps smaller than 3 eV, a 300 W xenon lamp and a filter are used to generate visible light. A solar simulator is also used as incident light when evaluating solar hydrogen evolution. Different types of reaction cells have been reported in the literature. Cells with two simultaneous semiconductors were employed in the 1970s and 1980s [39,69]. Single-junction cells have been reported to drive the hydrogen evolution reaction. However they are not satisfying for the overall water splitting due to insufficient photovoltage [67,68]. Multi-junction devices coupled with electrocatalysts could provide a large enough photo-voltage to drive water splitting [70]. A monolithic three-junction amorphous silicon photovoltaic cell coupled to cobalt phosphate and Ni-Zn-Mo tri-metal catalyst has been reported and exhibited an efficiency of 4.7% [92].

## 3. Photocatalytic Condition

Various parameters affect the photocatalytic activity of an inorganic photoconductor including surface chemistry, surface and junction defects [93], crystallinity, doping and deep traps, band edge positions, particle size, and morphology [94]. A variety of methods are tried for controlled synthesis of photocatalyst to tune these variables including hydrothermal [95], microwave assisted [96], surfactant assisted [97], and sonochemical [98] synthesis.

The synthesis parameters modify the activity of the catalyst especially when dealing with morphologically active nanoparticles and high surface area structures. Catalyst synthesis conditions including temperature, surfactants, concentration, and pH impact the structural features including crystal size, shape, and structure of the material [99]. Concentrations of building blocks in the solution affect nucleation and growth of the crystal structure, ultimately determining the activity. This is particularly tunable for co-catalytic systems where one-dimensional and two-dimensional structured materials have a geometric dependency. Copper oxide and zinc oxide core-shell nano wires [100], Cu_2_O/CuO heterojunction decorated with nickel [101], three dimensional branched cobalt-doped α-Fe_2_O_3_ nanorod/MgFe_2_O_4_ heterojunction [102], CuO nanoplates coupled with anatase TiO_2_ [103] are examples of geometrically active co-catalytic systems. For the synthesis of inorganic photocatalysts, pH along with hydrothermal temperature, treatment time, and solvent ratio control the morphology [104,105,106,107,108,109]. For BiVO_4_ solvent volume ratios of ethylene glycol over water (EG/H_2_O) ranging from 10/50 to 60/0 completely change the morphology of nanostructures. At 10/50 and 20/40, the FE-SEM images show lamellar shapes, with 20/40 making sheets thicker. Then at 30/30, 40/20, 50/10, and 60/0, leaf-like, bowknot-like, candy-like, and olive-like shapes were obtained. This is explained by higher viscosity of EG and its inhibitory effect on crystal growth thus allowing the nanocrystals to rotate and find a 3D structure to stabilize [110,111,112]. The morphology of BiVO_4_ is controllable with pH ranging from irregular microparticles at high pH to more uniform sized hollow structure microspheres at low pH [106].

### Surface and Band Structure

There are various studies indicative of surface chemical functionality, local structure and morphological characteristics of catalysts affecting the photocatalytic activity of the water splitting reaction. Surface chemical functionality is modified to protect against corrosion; deactivate destructive surface states; tailor band-edge positions; or selectively extract of carriers to improved catalytic activity [41,113]. Other than the increased catalytic activity via surface area, there is a trade-off between light absorption and carrier diffusion lengths which surface structure does influence. Increased surface area may lead to decreased photovoltage and increased surface recombination. Therefore careful understanding of loss mechanisms are required before surface modifications are employed [41].

Sheet-like morphologies exhibit higher light absorption than that of spherical morphologies for CuO. On reducing the crystallite size, band gaps shift towards lower energies [114]. One of the most recent examples is BiVO_4_ where the narrow band gap (2.4 eV) of the material and various possible morphologies has drawn research interest. Control and desirable structures, as well as morphology of BiVO_4_ for photocatalytic activities are necessary here [115,116]. Photocatalytic activity is shown to vary depending on the exposed facets of BiVO_4_ [117]. Synthesis of various types of BiVO_4_ morphologies has been studied including single crystal microspheres [118], heterophase microspheres [119], octahedra and decahedra [105], peanut-like and needle-like [107], star-like nanoplates [108], spindly hollow microtubes [120], leaf-like [121], hyperbranched-like [122], flower-like [123], porous olive-like [124], fusiform-like [116], and butterfly-like shapes [125].

Composites and multicomponent catalysts of BiVO_4_ have been studied with various morphologies; buckhorn-like BiVO_4_ with a shell of CeO_x_ nanodots [126], one-dimensional spindle-like BiVO_4_/TiO_2_ nanofibers [127], nest-like Bi_2_WO_6_/BiVO_4_ composite [128], flower-like monoclinic BiVO_4_/surface rough TiO_2_ ceramic fibers [129], and shuriken-shaped m-BiVO_4_/{001}-TiO_2_ heterojunction [130].

Band gap engineering is one of the main ways to increase the process efficiency where adjusting the layer thickness and sequence leads to the development of new electronic states [131]. Assuming junction materials to be A and B, junction architecture comes in three types: Type I is when the CB of material A is higher than that of material B while the VB of A is lower than B. Since electrons/holes tend to move downward/upward respectively for lower energy, they both get accumulated in material A. In type II, the VB and CB of Material A are both lower than those of Material B which leads to charge carrier separation. Type III is similar to type II with enhanced differences between CB’s and VB’s of the junction material [132]. Type II junction architecture is one widely used method to fabricate photocatlyst heterojunctions [133]. Enhanced activity due to charge carrier separation is reported for CdS/TiO_2_ [134,135] and CdS/ZnO [136,137] heterostructures. Nanostructuring of the junction films are important since excessive thickness would hinder efficient charge transfer. It is suggested that the film thickness be comparable with charge carrier diffusion length while being thick enough to significantly absorb light [41]. Nanostructuring of BiVO_4_/WO heterojunctions has led to near theoretical maximum photocurrent generation of BiVO_4_ material [138].

A recent study showed that band gap engineering techniques could allow photocathodes to carry out the water reduction reaction step of a PEC cell by using molecular beam epitaxy. Growing a wide band gap oxide of strontium titanate (SrTiO_3_), to a 4 nm thick layer acts as a protection layer for silicon as well as a tunneling junction for charge transport. The substrate being p-type silicon is matched with SrTiO_3_ lattice, so a perfect interface with very low density of defect can be fabricated. A maximum photocurrent density of 35 mAcm^−2^ was attained under a broad-spectrum illumination at 100 mWcm^−2^ as well as an open circuit potential of 450 mV [139]. Liao et al. synthesized cobalt(II) oxide (CoO) nanoparticles with shifted band edge position that could achieve solar-to-hydrogen efficiency of around 5% [140].

UV active semiconductors can be turned into visible light active materials by addition of cations and anions. Coupling wide and narrow band gap materials to get the right spectrum for full spectrum harvesting is utilized by co-catalysts such as CuO/ZnO [141]. Doping could favorably affect band gaps of photocatalysts due to successful band gap reduction of the photo-anode.

## 4. Photocatalyst Materials

### 4.1. Design and Description

**Figure 3:**
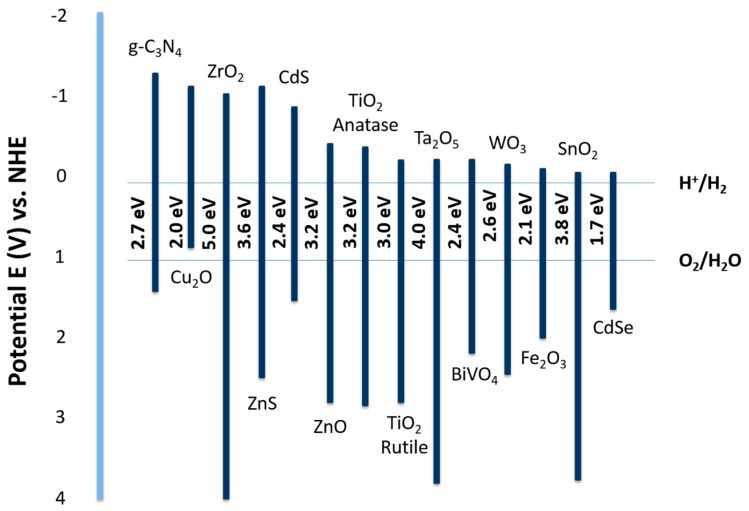
Band structure illustration of various semiconductors with respect of the redox potentials of water splitting. Figure adapted from reference [142] of Ong et al. work.

As previously mentioned, a suitable photocatalyst for overall water splitting should have a band gap of at least 1.23 eV with no photocorrosion. In terms of water splitting, high crystallinity and small particle size are desired to minimize the recombination of photo-generated electrons and holes. Metal oxides, sulfides, nitrides, and phosphates with d^0^ and d^10^ metal cations have been employed as water splitting catalysts. Group I, and Group II metals along with some lanthanides form perovskite materials can also be used to catalyze photochemical water splitting. The band structure of different types of semiconductors with respect of the redox potentials of water splitting are summarized in Figure 3. To improve solar energy efficiency, modification of photocatalysts by doping with some transition metal cations such as Ni^2+^, Cr^3+^, and V^5+^ can help to increase the visible light response. To prohibit the energy decreasing backward reaction of water splitting and increase the hydrogen production yield, suitable co-catalysts including RuO_2_, NiO, Au and Pt can be used. In this section, we will focus on heterogeneous photocatalysts including TiO_2_, metal oxides, metal sulfides, and metal nitrides.

### 4.2. TiO_2_

**Figure 4:**
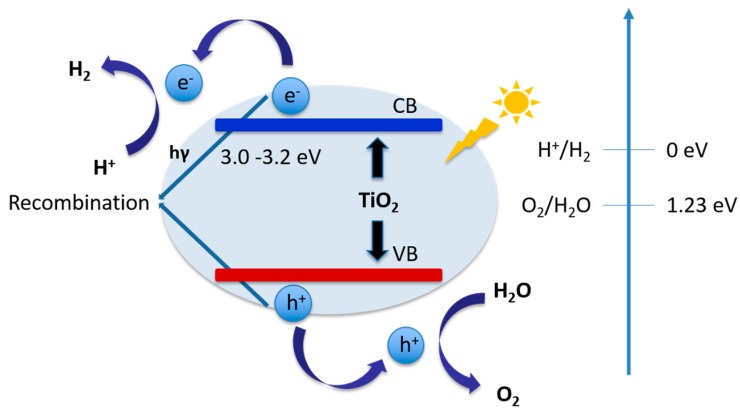
Schematic band gap diagram of TiO_2_. Figure adapted from references [4,149] of Moniz et al. and Miao et al. works respectively.

Since Fujishima and Honda first demonstrated that TiO_2_ was a promising photo-anode for UV light-driven photocatalytic water splitting [34], which has been widely studied in many photocatalytic reactions due to its chemical stability, low cost, environmentally friendly nature, and tunable electronic energy band gap [143,144,145,146,147]. Figure 4 shows a band gap illustration of TiO_2_. 

The valence band of TiO_2_ is more positive than $E_{ox}^{0}$ of O_2_/H_2_O (1.23 V vs. NHE, pH = 0), while the conduction band is more negative than $E_{red}^{0}$ of H^+^/H_2_ (0 eV vs. NHE, pH = 0) [148]. However, TiO_2_ materials suffer from two major drawbacks. One is the fast charge carrier recombination, which results in the release of unproductive energy. Another one is the inability to harvest visible light [149], since TiO_2_ can only be excited by UV light due to its wide band gap of 3.0–3.2 eV, which only covers 5% of the solar spectrum [14]. To enable visible light harvesting and prevent the recombination of photogenerated electron-hole pairs, proper modification should be performed. In this section, suitable modification methods will be introduced, including doping, making heterojunctions with other semiconductors or metals, and structural changes.

Doping TiO_2_ with other elements can change the optical properties and suppress the charge recombination adequately [150]. A variety of metals and non-metals have been doped into TiO_2_ materials. Anionic doping of TiO_2_ has been extensively reported by various dopant elements such us B, C, N, F, S, and Cl [151,152]. Luo and co-workers [153] have synthesized Br and Cl co-doped TiO_2_ via a hydrothermal method, in which a titanium chloride is used as titanium source and incorporated bromide by hydrobromic acid. The unique Br and Cl-doped TiO_2_ exhibits an extended light absorption into the visible light region, in which the non-metal dopants were proven to be the key factor to narrow the band gap. The resulting material showed an enhanced solar light-induced water splitting activity.

The other alternative method to extend the photocatalytic activity of TiO_2_ to visible light region is to dope this material with carbon. For instance, Faria et al. have reported doping of TiO_2_ with carbon nanotubes (CNTs) [154]. Although different mechanisms have been proposed to explain this enhancement, the mechanism of synergic effect of carbon on TiO_2_ remains unclear. Three mechanisms have been explored to describe the synergetic effect of carbon on TiO_2_. The first possible mechanism is carbon can play the role of an electron sink, which can effectively prevent the recombination process [155]. Another mechanism proposes carbon as a photosensitizer, which can pump electrons into TiO_2_ conduction band [156]. Besides the proposed mechanisms, carbon can also act as template to disperse the TiO_2_ particles and hinder the agglomeration of TiO_2_ nanoparticles [157].

**Figure 5:**
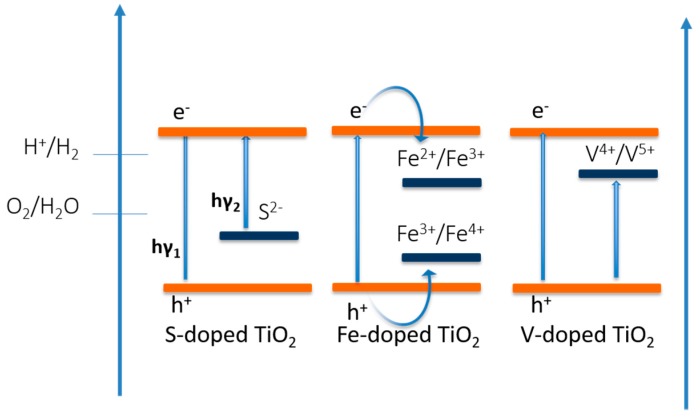
Schematic band gap alignment of S-doped, Fe-doped, and V-doped TiO_2_. Figure adapted from reference [158] of Babu et al. work.

Unlike non-metal ion doping, metallic dopants usually introduce additional energetic levels in the band gap, which reduce the energy barrier and induce a new optical absorption edge [158,159]. Piskunov et al. [152] have reported enhancement in photocatalytic water splitting activity of Fe-doped TiO_2_, where Fe^2+^/Fe^3+^ acts as electron-trap centers and Fe^3+^/Fe^4+^ acts as hole-trap centers. Luo et al. [97] have shown at vanadium doping shifts the absorption band to the visible region and the V^4+^/V^5+^ pair efficiently traps the electrons and holes, which suppress the recombination of electron and holes. Figure 5 represents the schematic band alignment of doped TiO_2_ semiconductors.

**Figure 6:**
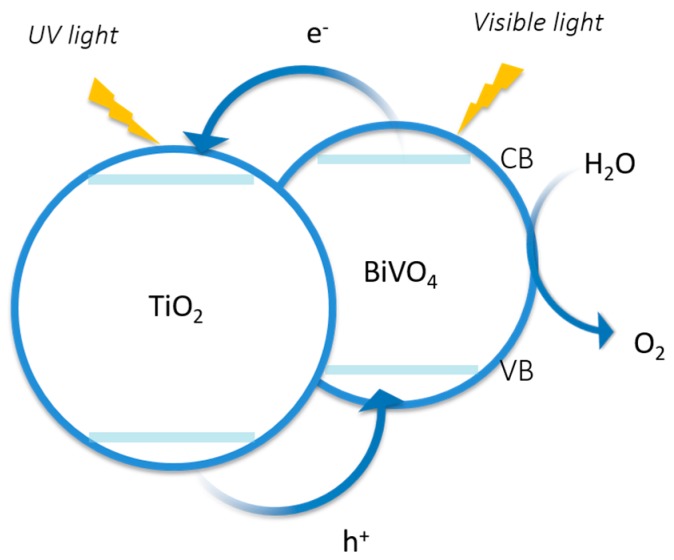
Schematic band gap alignment of TiO_2_/BiVO_4_ heterojunction. Figure adapted from references [4,161] of Moniz et al. and Resasco et al. works respectively.

The formation of a semiconductor-semiconductor heterojunction can decrease the charge recombination rate by yielding long-lived electron-hole pairs [160]. Proper band alignments allow charge transfer from one semiconductor to another [4]. Resasco et al. [161] have reported a TiO_2_/BiVO_4_ host-guest photo-anode system, in which TiO_2_ acts as an electron acceptor, and BiVO_4_ serves as a visible light capturer. Due to the good electron affinity of TiO_2_ and the small optical band gap of BiVO_4_ (2.5 eV), the resulting heterojunction as a photo-anode performed better than bare TiO_2_ or BiVO_4_ (Figure 6).

Using a sacrificial agent helps TiO_2_ in performing either water oxidation or reduction. The sacrificial agent reacts with one of the charge carriers while the other carrier is in charge of either oxygen or hydrogen production. Typically, sacrificial agents such as methanol, ethanol and ethylene glycol, which have, lower oxidation potentials than water are used to inhibit the electron hole pair recombination in TiO_2_ [162]. In another scenario the valence band energy level of one semiconductor is higher than the other while the conduction band energy level is lower than the other semiconductor. As a result of this band gaps alignment of two semiconductors, a charge separation occurs and recombination process decreases [163].

In a metal-semiconductor heterojunction structure, noble metals, such as Au, Pt, Pd, Ru, have been reported to trap photogenerated electrons due to their significant role as electron sinks. Among noble metals, Au has been studied as the preferred co-catalyst for photocatalytic hydrogen production due to its high affinity towards photo-generated electrons, high resistance to oxidation, less activity towards the side reactions of hydrogen production, and the existence of surface plasmon resonance [164,165,166]. Wu et al. [167] have investigated the anisotropic growth of TiO_2_ onto Au nanorods, which achieved an enhanced visible light-induced hydrogen production. The close contact between TiO_2_ and Au facilitated the generation of surface plasmon resonance induced electrons. By engineering the structure, the performance of hydrogen evolution under visible light irradiation could be improved.

**Figure 7:**
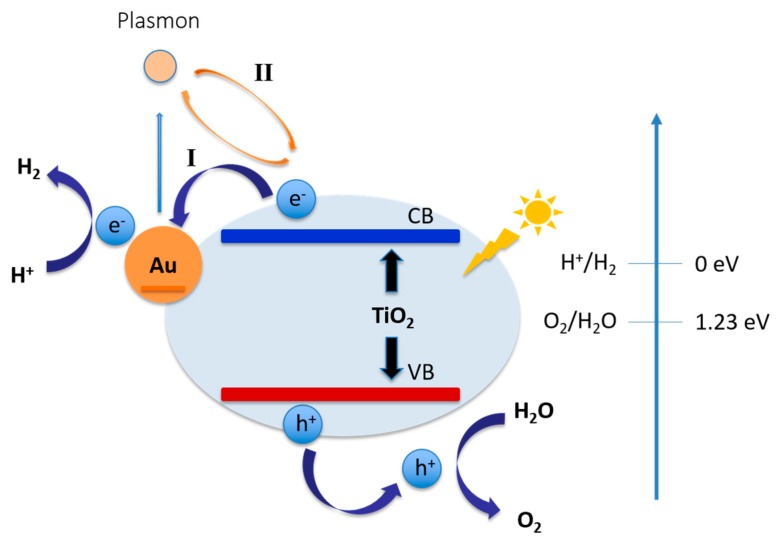
Schematic illustration of heterojunction between Au nanoparticles and TiO_2_ semiconductors. Pathway I shows the extraction of photo-generated electron from TiO_2_ conduction band to Au NPs. Pathway II shows the coupling of exciton of TiO_2_ and surface plasmon of Au. Figure adapted from references [150,168] Chen et al. and Dutta et al works respectively.

Figure 7 shows the electron transfer pathways between Au nanoparticles and TiO_2_ semiconductors. Another method to facilitate the photocatalytic water splitting process in the TiO_2_ system is by structural modification. The structure of TiO_2_ has a significant effect on the photocatalysis performance. Li et al. [169] have reported that amorphous TiO_2_ with more defects suffers from a faster charge recombination rate than high crystalline TiO_2_. Other than crystallinity, the mesoporous structure of TiO_2_ also plays a key role in the study of photo-electrode. Zheng et al. [170] have found at the existence of a mesoporous structure favors the rapid diffusion of products and suppresses the electron/hole recombination. Also, the morphology of the photocatalyst has a major effect on the photocatalytic activity [4]. 1-D TiO_2_ forms such as nanotubes [152], nanowires [171], and nanofibers [172] have been studied and show improved photocatalytic activity. Tuning of morphology has attracted considerable attention due to the change in material morphology that can alter charge carrier diffusion pathways. Therefore, to improve the photocatalytic hydrogen evolution efficiency of TiO_2_, modification of its structure is highly relevant.

Among various strategies to overcome fast charge recombination which leads to low photocatalytic efficiency [173,174] plasmonic photocatalysis is the most promising approach to promote charge separation and visible light absorption [175,176]. Nanoparticles of Au [177,178,179,180], Ag [181] and Pd [182] have been applied to improve visible and near-infrared (NIR) absorption and generate surface plasmon resonance (SPR) hot electrons [176]. The plasmonic properties of metallic nanoparticles (normally Au and Ag) are very attracting due to their ability to promote catalytic reactions. Oscillation of the conduction band in the plasmonic structures results is localized surface plasmonic resonance (LSPR) which finally leads to hot electron generation through non-radiative decay of plasmons. These electrons assist catalytic reactions [183]. Wang et al. have discussed two mechanisms that surface plasmon can enhance the electron-hole formation and separation: (1) local electromagnetic field enhancement (LEMF) in order to enhance interband transition rate through strong local field; and (2) resonant energy transfer (RET) from the plasmonic dipoles to the e–h pairs in the semiconductor through a near-field electromagnetic interaction [184]. Composition, shape and environment of noble metal nanoparticles significantly influenced on their surface plasmon resonance (SPR) property [185]. Among the various Au nanoparticles shapes [186,187,188,189], nanorods have been used for H_2_ evolution. To make the most of the charge separation of hot electrons, Au nanorods are usually interfaced with efficient electron acceptors (e.g., TiO_2_) [167]. Moreover, Au triangular nanoprisms (TNPs) have shown different modes of SPR [185]. These anisotropic structures are significantly crucial for the hot electron transfer [190].

### 4.3. Metal Oxides

Other than TiO_2_, a number of other representative metal oxides (such as Fe_2_O_3_, WO_3_, ZnO, Cu_2_O, Al_2_O_3_, Ga_2_O_3_, Ta_2_O_5_, CoO and ZrO_2_) have also been widely studied due to their stability in aqueous solution and their low cost. However, most metal oxides suffer from large band gaps limiting their ability to absorb visible light.

In a typical metal oxide, the valence band and conduction band have O 2p and metal s character and therefore relatively ionic bonded materials have a large band gap [46]: ZnO (3.4 eV) [191], Ga_2_O_3_ (4.5 eV) [192], Al_2_O_3_ (8.8 eV) [193]. Using transition metal cations with d^n^ electronic configurations may help overcome this issue, Fe_2_O_3_ (∼2.0 eV) [46,194] and Co_3_O_4_ (∼1.3 eV) [195] have increased light absorption but lack efficient charge carrier transfer due to small polaron dominated conductivity and associated high resistivity [196,197]. Using post-transition metals like PbO (2.1 eV) [191,196], SnO (2.4 eV) [197,198] and Bi_2_O_3_ (2.5 eV) [191,199] with occupied s band leads to better photogeneration of charge carriers however they are indirect semiconductors where optical absorption band edges vary with the square root of photon energy leading to a less efficient carrier extraction process [46]. Therefore ternary metal oxide compounds have been investigated to overcome these limitations such as Bi_20_TiO_32_ [200], SnNb_2_O_6_ [201] and BiVO_4_ [46,50]. BiVO_4_ has been investigated for having both a low band gap (2.4–2.5 eV) and reasonable band edge alignment for the water redox potentials [202]. Both n- and p-type semiconducting properties have been recorded by BiVO_4_ as well as high photon-to-current conversion efficiencies (>40%) [203].

Mishra et al. [204] have reported that Fe_2_O_3_ as photocatalytic materials has a proper band gap of 2.2 eV, which allows photon absorption under visible light irradiation. However, severe bulk recombination has limited the usage of Fe_2_O_3_. Some mechanistic studies also have been conducted for water oxidation and reduction reactions. Haghighat et al. [205] have studied the mechanism of water oxidation on iron oxide photocatalysts through evaluating the electron-transfer by changing the pH and potential space during the process. Morales-Guio et al. have designed an oxidatively electrodeposited optically transparent photocatalyst of amorphous iron-nickel oxide (FeNiO_x_) for the oxygen evolution reaction [206]. It was demonstrated that low loading of FeNiO_x_ and its high activity at low over potential was achieved in unassisted water splitting with solar-to-hydrogen conversion efficiencies more than 1.9% and ∼100% Faradaic.

Similar to Fe_2_O_3_, WO_3_ has been considered as a potential photo-anode material for its suitable valence band position, which favors a high onset potential for water oxidation. Elsewhere, Amer et al. [207] have reported ZrO_2_ modification by deposition of thin layers of ZrN on ZrO_2_ nanotubes, to prepare core-shell structures for the photo-anode activated under visible light. However, Moniz et al. [4] found that the main drawback of WO_3_ is its instability toward anodic photocorrosion. These low E_g_ materials (e.g., Fe_2_O_3_ and WO_3_) can be modified including doping with metal cations or by forming heterojunction structures with other semiconductors [158]. Sivula et al. [208] have demonstrated a WO_3_/Fe_2_O_3_ photo-anode for water oxidation by using WO_3_ as a host scaffold to support Fe_2_O_3_ thin layer, due to the suitable band gap alignment between WO_3_ and Fe_2_O_3_ allowing fast electron transfer at the interfaces of host/guest. They found an increase in the photon absorption efficiency and a higher surface area of Fe_2_O_3_, resulted in a higher activity for water oxidation.

Cobalt oxide (CoO) also shows photocatalytic activity toward H_2_ evolution [140,209]. Liao et al., have reported CoO nanocrystals as photocatalyst for water splitting under visible light [140]. However, short lifetime and fast deactivation of CoO nanoparticles limit their usage as photocatalyst for hydrogen evolution. As the morphology of nanostructures can influence on the band-edge positions of material [210], designing CoO with different morphology such as nanotubes or nanowires could provide more efficient photocatalyst materials. Zhan et al. developed CoO nanowires on the carbon fiber papers with hydrogen generation rate of 81.3 μmol·g^−1^·h^−1^, which indicate higher chemical stability in comparison with CoO nanoparticles [209].

SrTiO_3_ (STO) has also been widely used for hydrogen production as a solid-state photocatalyst with a band gap of 3.2 eV, which has been explored for the overall water splitting under UV light irradiation. Since STO is active toward water splitting only in the UV region, the solar to hydrogen conversion (STH) is low. Doping methods enhance the quantum efficiency of SrTiO_3_ in the visible light region [211,212,213]. Domen et al. have recently reported the photocatalytic behavior of SrTiO_3_ in the overall water splitting reaction can significantly be improved by flux-mediated Al doping. Doping Al in SrTiO_3_ has improved the photocatalytic activity by 30% at 360 nm. In another study, SrTiO_3_ is doped with a small amount of Rh to provide a donor level in the band gap region of SrTiO_3_: Rh. This prevents the charge recombination and subsequently facilitates the hydrogen production (2223 μmol·h^−1^·g^−1^) in comparison to pure STO [214].

Tantalum oxide (Ta_2_O_5_) has been an attractive semiconductor for photocatalytic water splitting [215,216,217,218]. Due to the wide band gap of Ta_2_O_5_ (about 4 eV), it is required to narrow the band gap with some techniques such as doping with foreign ions. Lu et al. have described Ta_2_O_5_ nanowires as an active photocatalysts which generated hydrogen with the rate of 214 mmol·g^−1^·h^−1^ under Xe lamp irradiation without any cocatalyst [215]. They have discussed that Ta_2_O_5_ nanowires with low dimensional structures provide higher surface area with favorable carrier transport to harvest light for H_2_ production. Very recently, Zhu et al. have reported gray Ta_2_O_5_ nanowires which were modified by aluminum reduction to improve electron density and photoelectrochemical water splitting properties of the material [218].

### 4.4. Metal Sulfides

CdS and ZnS are the most studied metal sulfide photocatalysts in the past decades [148]. Compared to metal oxide semiconductors, CdS with narrower band gap (~2.4 eV) is considered promising as a visible-light-driven photocatalyst for water splitting [99]. However, as a result of rapid recombination of photogenerated electrons and holes, bare CdS semiconductors usually show low hydrogen production rates [164]. Moreover, high activity of CdS under light irradiation leads to the corrosion of semiconductors [163]. To circumvent this problem, CdS materials can be coupled with other noble metals as co-catalyst or form a heterojunction structure with other semiconductors [219]. In such a case, the photogenerated electrons on the conduction band of CdS can be transferred to electronic levels of noble metals or be delocalized and transferred between the conduction bands of semiconductors. Huang et al. [220] have established a hollow bimetallic sulfide material with a very narrow band gap. The bimetallic metal sulfides exhibit a hydrogen production rate comparable to Pt when sensitized by Eosin Y dye or coupled with TiO_2_ and C_3_N_4_ semiconductors. Nickel sulfide, in particular has proven to be tremendously useful in raising the activity of semiconductors when use as a co-catalyst along with TiO_2_, CdS, and g-C_3_N_4_ [221]. Unlike CdS, wide band gap ZnS (3.6 eV), responds weakly to visible light [222,223]. Efforts have been made to improve the photoactivity of ZnS for hydrogen evolution. Li et al. [224] have reported highly active Zn_1-x_Cd_x_S solid solution systems for visible-light induced hydrogen production. The band gaps of the solid solution photocatalysts can easily be tuned by varying the Zn/Cd molar ratios. Compared to bare ZnS with a large band gap, the narrower band gap of the resulting solid solution photocatalyst favors the absorption of photons under visible light irradiation. Metal oxides with large band gaps provide higher stability for the composite material [225]. Some of the metal oxides that have been proposed to combine with CdS are TaON, TiO_2_ and ZnO [226,227]. 

Different nanostructures of carbon can also be combined with CdS to promote catalytic behavior towards water splitting by preventing the charge recombination process. Due to the high conductivity of the carbon nanostructures, any contact between CdS and carbon can substantially improve the charge separation and subsequently the catalytic behavior of nanocomposites will be enhanced. Different strategies have been taken to synthesize the carbon-based CdS from simple mixing of carbon and CdS to in-situ growing of the CdS at the surface of graphene oxide using oxygen moieties as the template [228]. WS_2_-Au-CuInS_2_ has also been developed for photocatalytically H_2_ production by insertion of gold nanoparticles between of WS_2_ nanotubes and CuInS_2_ (CIS) nanoparticles [229]. Introducing Au nanoparticles led to significant enhancement of light absorption. Moreover, H_2_ evolution efficiency has been reported as the highest one for WS_2_-Au-CIS due to the more rapidly photogenerated carrier separation from the type II band structures and the localized surface plasmonic resonance (LSPR) effect from the Au nanoparticles.

### 4.5. Nitrides

To efficiently harvest solar light, nitrides and oxynitrides can be applied as photocatalysts for water splitting [230]. The 2p orbitals corresponding to nitrogen in nitrides have higher energy than analogous orbitals of oxygen in metal oxides. Consequently, in nitrides lower energy is needed to excite electrons to the conduction band. A solid solution of GaN and ZnO has been considered as a promising photocatalyst for water oxidation [231]. Although GaN and ZnO are poor visible light absorbers due to their large band gaps, after mixing (Ga_1-x_Zn_x_)(N_1-x_O_x_) will have new electronic states that considerably reduce the band gap [232]. The other well-known catalyst for water splitting is a perovskite-like mixed oxide of NaTaO_3_ and SrTiO_3_, which has a 50% quantum yield for the 280 nm-light [233]. Replacement of one of the oxygens in these oxides with nitrogen leads to a shift in the absorption edge toward higher wavelengths (600 nm), which improves their photocatalytic activity under visible light.

Tantalum nitride (Ta_3_N_5_) also has been identified as an active photocatalyst for water splitting. In 2013, Zhen et al., reported a template-free synthesis of Ta_3_N_5_ nanorod which was modified with Co(OH)_x_ to be used as anode for photoelectrochemical cell for water splitting [234]. Elsewhere, Ta_3_N_5_ has been modified by partial substitution with Mg^2+^ and Zr^4+^ which led to apparent decrease in onset potential for PEC water oxidation [235]. Such compositional modification could apply to other semiconductors in order to enhance photocatalytic activity.

Other than the oxynitrides, graphitic carbon nitrides (g-C_3_N_4_) also have been utilized as photocatalysts to produce hydrogen due to their narrow band gap of 2.7 eV that has a conduction band shallower than hydrogen evolution potential and a valence band potential deeper than the reversible oxygen evolution potential. g-C_3_N_4_ could produce hydrogen from water under visible light (<540 nm) in the presence of a sacrificial agent (oxidizing agent) without the aid of any noble metal. However, pristine g-C_3_N_4_ shows a low affinity towards photocatalytic reactions. Wang et al. have reported for the first time a graphitic semiconductor that was synthesized from cyanamide [236] which showed an absorption edge in the visible light region and steady hydrogen production over 75 h. Band gap engineering of g-C_3_N_4_ has been reported to enhance the photocatalytic properties through a non-metal (i.e., S, F, B, P) [237] and metal doping (i.e., Pt, Pd, Fe, Zn, Cu) [238] strategy. Moreover, the charge separation in g-C_3_N_4_ can also be enhanced by applying conductive graphene, carbon nanotubes, and reduced graphene oxide at the interface with g-C_3_N_4_ [239]. Martin et al. have shown nature-inspired semiconductor systems among which the most efficient system consist of g-C_3_N_4_ and WO_3_ for hydrogen and oxygen evolution (21.2 and 11.0 μmol·h^−1^·g^−1^, respectively) under visible light for 24 h [240]. They conclude that g-C_3_N_4_ can be considered as a multifunctional photocatalyst with the ability of application in PEC cells or coupled solar systems.

Recently, Suib et al. have reported a metal-free carbon-based nanocomposite for the hydrogen evolution under visible light using various precursors which resulted in different morphologies, band gaps and consequently different photocatalytic activities. Hybridized g-C_3_N_4_ with nitrogen doped graphene quantum dots showed a higher photocatalytic activity for the nanocomposite [241]. 

## 5. Theoretical Modeling of Photocatalytic Water Splitting

Theoretical studies concern various aspects [242] of photocatalytic reactions such as light absorption [243], electron/hole transport [244,245], band edge alignment of semiconductors [246,247] and surface photoredox chemistry [248]. Density Functional Theory (DFT) [249,250] is extensively used as a theoretical method to predict and understand the electronic structure of materials due to high accuracy, predictive power, modest computational cost [251] and reproducibility [252]. However, one of the major shortcomings of DFT has been the inaccurate prediction of band gaps. This is because DFT formulation lacks a proper description of self-interaction and correlation terms. Pragmatic approaches involve hybrid functional or addition of electron repulsion to selected localized orbitals [242].

Hybrid functionals have better accuracy for band gap prediction and the position of the excited states but are computationally demanding compared to standard exchange and correlation functional forms [253]. The proper way to tackle the band gap problem is through many-body perturbation theory (MBPT) which has a long-standing record of success [254,255]. This approach although being computationally expensive, provides a standard for comparative studies to develop new methods [253]. One more recent approach known as TB09 has been proposed using a modified version of the Becke-Johnson exchange potential [256] combined with an LDA correlation [257]. This method along with variations have been shown to be some of the most accurate approaches found in the literature to this date relative to computational cost [253,258].

Computational methods are especially helpful for prediction of impurity states induced by dopants in tuning band gaps in photocatalytic systems such as TiO_2_ [242]. Time-dependent density functional theory (TD-DFT) is not widely used, and the numbers of studies implementing these methods are cluster-based models [4].

Besides predictive power, theoretical and computational tools are capable of advancing our understanding of various aspects of states. For example, for BiVO_4_ comprehensive studies investigated the band structure and density of states [46], electron/hole generation, and migration and energy profiles of surface reactions [259]. For BiVO_4_ photoexcited electrons and holes are driven to different crystal facets [260]. These findings were obtained by comprehensive computational studies that showed that compared to (011) facets, the (010) facets have lower absorption beyond 420 nm, better transport of electron/holes, more favorable water absorption, and lower potential energy surfaces for OER [259]. Theoretical studies like that would lead to rational improvement of band structure and morphological design of the photocatalytic material. 

With advances in accuracy and eventual decrease of computational costs, high-throughput computational screening is going to be an emerging field. This is going to help with choosing the optimal components hence slashing the time of discovery of new materials to a fraction of what now it used. Experimental rapid screening is reported by scanning electron microscopy [52], and multiplexing counter electrode [261] for photocatalytic material discovery. However, computational screening studies currently on record related to photoactive materials are rare and very recent, which highlights the potential of impactful research that could soon emerge in this area [262,263,264].

## 6. Conclusions

Hydrogen production from solar energy using photocatalytic active materials has been considered as one of the most promising steps towards generating clean and renewable alternatives for fossil fuels. In order to use solar energy more efficiently, different approaches have been employed to shift photocatalyst activity towards the visible range while retaining stability and efficiency. TiO_2_ as the pioneer photocatalyst also has some limitations such as wide band gap, high hydrogen overpotential, and rapid recombination of produced electron-hole pairs which have been addressed by various methods including doping, coupling with carbon, noble metal deposition, using dyes, and surface modifications. Other metal oxides such as iron oxide, zinc oxide, copper oxide also have been discussed as well as metal sulfides including cadmium sulfides, and zinc sulfides. In addition, nitrides and nanocomposite materials that have been used as photocatalysts for water splitting have been reviewed.

The current outlook of efficient water splitting relies on an innovative design of photocatalytic materials. Recent studies on heterojunction photocatalysts have shed light on the nature of charge transfer. Heterojunctions involving carbon based material is believed to be one of the feasible future routes to efficient photocatalyst design [4]. The architecture of the heterojunction directly influences the activity and could potential lead to great improvements [265,266]. The future direction of photocatalytic water splitting is more focused on development of an efficient photoanode with band edges that match the redox potentials of water and with rapid charge-transfer activity under visible light while maintaining chemical and physical stability [267]. Theoretical and computational models could help us understand the electronic density of states and band structure and therefore point towards a rational design of photocatalysts [46]. Computational high throughput screening is an emerging field that will be utilized in material selection and junction design to yield optimized band structures.

## Figures and Tables

**Table 1:** Recent visible light active photocatalysts for water splitting.

Photocatalysts	Band Gap (eV)	Illumination	Hydrogen Production	Ref.
Pt, Cr, Ta Doped TiO_2_	N/A	Visible light (>420 nm)	11.7 μmol·h^−1^·g^−1^	[71]
Cu-Ga-In-S/TiO_2_	N/A	300 W Xe arc lamp (385–740 nm)	50.6 μmol·h^−1^	[72]
1 wt.%Pt/C-HS-TiO_2_	2.94	Visible light	5713.6 μmol·h^−1^·g^−1^	[73]
Platinized sub-10 nm rutile TiO_2_ (1 wt.% Pt)	2.7–2.9	Xe lamp (PLS-SXE, 300–2500 nm) with (UVREF: 320–400 nm, ca. 83 mW·cm^−2^; UVCUT400: 400–780 nm, ca. 80 mW·cm^−2^)	932 μmol·h^−1^·g^−1^ visible light 1954 μmol·h^−1^·g^−1^ simulated solar light	[74]
Rh- and La-codoped SrTiO_3_	N/A	300 W Xe lamp fitted with a cutoff filter (λ > 420 nm)	84 μmol·h^−1^	[75]
Cu_1.94_S-Zn_x_Cd_1−x_S (0 ≤ x ≤ 1)	2.57−3.88	visible-light irradiation (λ > 420 nm)	7735 μmol·h^−1^·g^−1^	[76]
MoS_2_/Co_2_O_3_/poly(heptazine imide)	N/A	visible light irradiation	0.67 μmol·h^−1^	[77]
Bi_4_NbO_8_Cl	2.4	visible light	6.25 μmol·h^−1^	[78]
CdS nanorod/ ZnS nanoparticle	N/A	visible light irradiation (>420 nm)	239,000 μmol·h^−1^·g^−1^	[79]
Ni/CdS/g-C_3_N_4_	N/A	300 W Xe lamp (≥420 nm)	1258.7 μmol·h^−1^·g^−1^	[80]
CdS/WS/graphene	N/A	visible light irradiation (>420 nm)	1842 μmol·h^−1^·g^−1^	[81]
V-doped TiO_2_/RGO	N/A	visible light irradiation	160 μmol·h^−1^	[82]
Pt/g-C_3_N_4_ Conjugated Polymers	2.56	visible light irradiation (>420 nm)	1.2 μmol·h^−1^	[83]
Au–TiO_2_ Nanohybrids	N/A	Vis-NIR irradiation (>420 nm )	647,000 μmol·h^−1^·g^−1^	[84]
SrTiO_3_:La,Rh/Au/BiVO_4_:Mo	N/A	300 W Xe lamp fitted with a cutoff filter (λ > 420 nm)	90 μmol·h^−1^	[85]
CoOx-B/TiO_2_-TaON	N/A	150 W Xe Lamp arc (>400 nm)	40 μmol·h^−1^	[86]
MoS_2_/CuInS_2_	N/A	300 W Xe lamp fitted with a cutoff filter (λ > 420 nm)	202 μmol·h^−1^·g^−1^	[87]
Copper-Organic Framework; H_2_PtC_l6_	2.1	UV-Visible irradiation	30 μmol·h^−1^·g^−1^	[88]
